# Data from quantitative proteomic analysis of lung adenocarcinoma and squamous cell carcinoma primary tissues using high resolution mass spectrometry

**DOI:** 10.1016/j.dib.2018.06.035

**Published:** 2018-06-22

**Authors:** Aafaque Ahmad Khan, Kiran Kumar Mangalaparthi, Jayshree Advani, T.S. Keshava Prasad, Harsha Gowda, Deepali Jain, Aditi Chatterjee

**Affiliations:** a Institute of Bioinformatics, 7th floor, Discoverer building, International Tech Park, Bangalore 560066, India; b School of Biotechnology, Kalinga Institute of Industrial Technology, Bhubaneswar 751024, India; c School of Biotechnology, Amrita Vishwa Vidyapeetham, Kollam 690525, India; d Manipal Academy of Higher Education, Manipal 576104, India; e YU-IOB Center for Systems Biology and Molecular Medicine, Yenepoya University, Mangalore 575018, India; f Department of Pathology, All India Institute of Medical Sciences (AIIMS), Ansari Nagar, New Delhi 110029, India

**Keywords:** Lung adenocarcinoma, Lung squamous cell carcinoma, iTRAQ labeling, Proteomics

## Abstract

Lung cancer is the leading cause of preventable death globally and is broadly classified into adenocarcinoma and squamous cell carcinoma. In this study, we carried out mass spectrometry based quantitative proteomic analysis of lung adenocarcinoma and squamous cell carcinoma primary tissue by employing the isobaric tags for relative and absolute quantitation (iTRAQ) approach. Proteomic data analyzed using SEQUEST algorithm resulted in identification of 25,998 peptides corresponding to 4342 proteins of which 610 proteins were differentially expressed (≥ 2-fold) between adenocarcinoma and squamous cell carcinoma. These differentially expressed proteins were further classified by gene ontology for their localization and biological processes. Pathway analysis of differentially expressed proteins revealed distinct alterations in networks and pathways in both adenocarcinoma and squamous cell carcinoma. We identified a subset of proteins that show inverse expression pattern between lung adenocarcinoma and squamous cell carcinoma. Such proteins may serve as potential markers to distinguish between the two subtypes. Mass spectrometric data generated in this study was submitted to the ProteomeXchange Consortium (http://proteomecentral.proteomexchange.org) via the PRIDE partner repository with the dataset identifier PXD008700.

**Specifications Table**

**Table:** 

Subject area	Biology
More specific subject area	Non-small cell lung cancer proteomics
Type of data	Mass spectrometry raw files, msf result files, Excel tables, Figure
How data was acquired	Orbitrap Fusion mass spectrometer (Thermo Scientific, Bremen, Germany), Proteome Discoverer (version 2.1.0.81) software suite (Thermo Fisher Scientific, Bremen, Germany) and SEQUEST algorithm with NCBI RefSeq human protein database (Version 75).
Data format	Raw and analyzed data
Experimental factors	Primary tissues were collected from lung adenocarcinoma and squamous cell carcinoma tissues
Experimental features	Quantitative proteomic analysis of lung adenocarcinoma and squamous cell carcinoma tissues
Data source location	Bangalore
Data availability	Output data in excel format is available here and raw data is available via a web application (ProteomeXchange) Consortium (http://proteomecentral.proteomexchange.org) via the PRIDE partner repository with the dataset identifier PXD008700.

**Value of the data**

• This data reveals protein expression pattern associated with lung adenocarcinoma and squamous cell carcinoma tissues
• Differentially expressed proteins between lung adenocarcinoma and squamous cell carcinoma may serve as potential biomarkers to distinguish these 2 subtypes
• The data provides information about biological processes and pathways enriched in lung adenocarcinoma and squamous cell carcinoma

## 1 Data

**Fig. 1:**
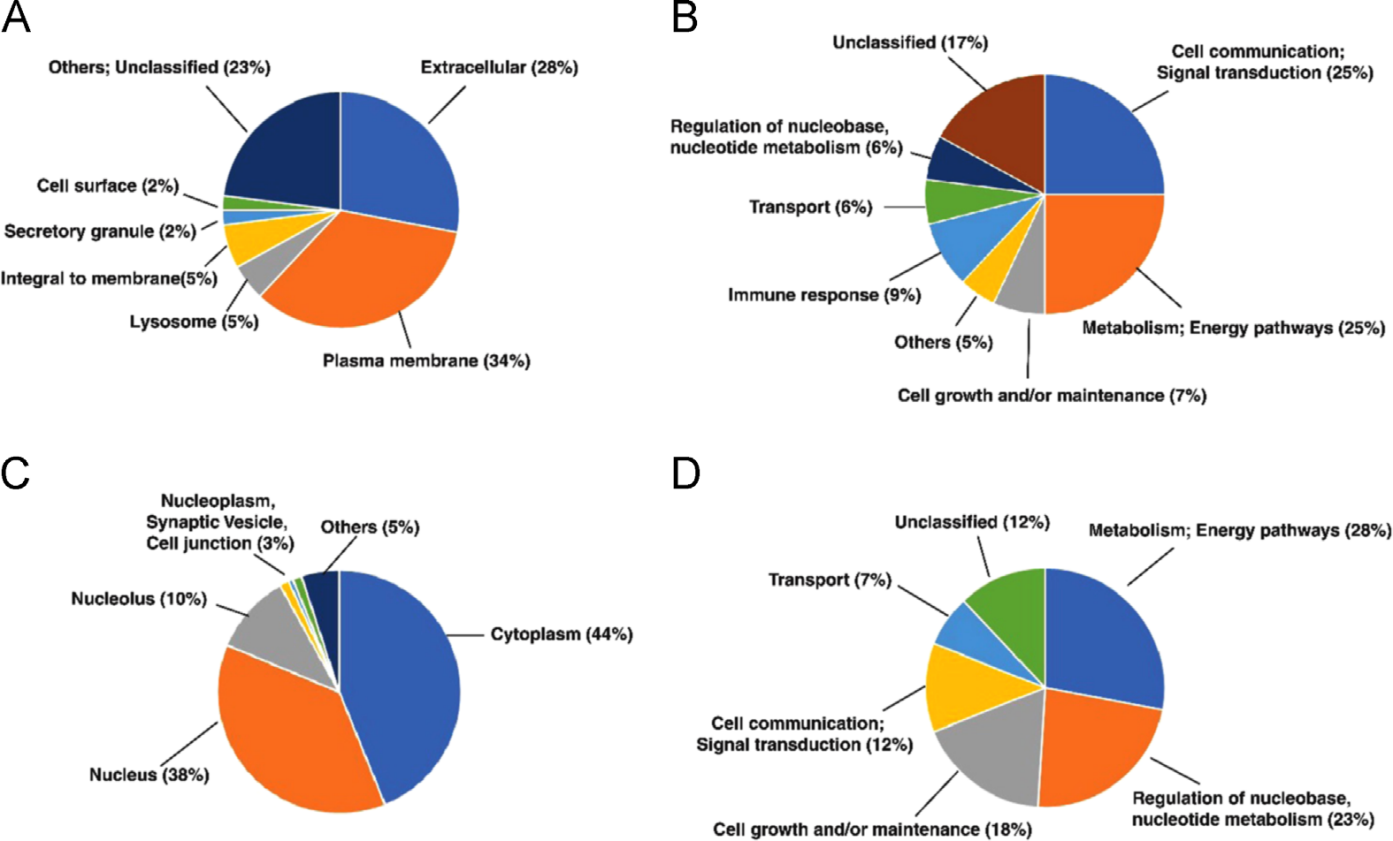
Gene ontology-based annotation of differentially expressed proteins identified in lung adenocarcinoma and squamous cell carcinoma samples. (A) Classification of overexpressed proteins from lung adenocarcinoma samples (downregulated in squamous cell carcinoma) based on subcellular localization. (B) Classification of overexpressed proteins from lung adenocarcinoma samples (downregulated in squamous cell carcinoma) proteins based on molecular function. (C) Classification of downregulated proteins from lung adenocarcinoma samples (overexpressed in squamous cell carcinoma) based on subcellular localization. (D) Classification of downregulated proteins from lung adenocarcinoma samples (overexpressed in squamous cell carcinoma) proteins based on molecular function.

This dataset contains raw and processed data following LC-MS/MS analysis of lung adenocarcinoma and squamous cell carcinoma primary tissues. The processed data contains 25,998 peptides corresponding to 4342 proteins. All proteins and peptides identified in this study are listed in Supplementary tables S1 and S2 respectively. We identified 610 proteins that are differentially expressed between adenocarcinoma and squamous cell carcinoma samples (≥ 2-fold). These differentially expressed proteins were further classified by gene ontology for their localization and biological processes **(**Fig. 1 A-D**)**. Further analysis of differentially expressed proteins revealed distinct alterations in pathways **(**Tables 1A**-**1B**)** and networks **(**Tables 2A-2B**)** in both adenocarcinoma and squamous cell carcinoma samples.

**Table 1A:** Top 5 canonical pathways enriched in upregulated proteins from lung adenocarcinoma samples (downregulated in squamous cell carcinoma).

**Canonical Pathway**	**P-value**	**Overlap (%)**
Leukocyte extravasation Signaling pathway	2.8E-13	12.6%
Fcγ receptor mediated phagocytosis	1.05E-10	17.2%
IL-8 Signaling pathway	1.2E-9	10.7%
Complement System	2.53E-9	27.8%
Production of nitric oxide and ROS in macrophages	3.08E-8	9.8%

**Table 1B:** Top 5 canonical pathways enriched in downregulated proteins from lung adenocarcinoma samples (upregulated in squamous cell carcinoma).

**Canonical Pathway**	**P-value**	**Overlap (%)**
Glutathione mediated detoxification	2.36E-6	20.8%
Glutathione redox reactions I	4.55E-5	18.2%
NRF2 mediated oxidative response	7.1E-5	4.7%
Methylglyoxal Degradation	2.08E-4	23.1%
Garanazyme A Signaling	4.81E-4	17.6%

**Table 2A:** Top 5 enriched networks in upregulated proteins from lung adenocarcinoma samples (downregulated in squamous cell carcinoma).

Network	Score
Cellular function and maintenance, Inflammatory response, Cellular movement	42
PTM, Protein degradation, Protein synthesis	32
Lipid metabolism, Small molecule biochemistry, Cell death and survival	32
Cellular movement, Cell to cell signaling and interactions	30
Lipid metabolism, Molecular transport	30

**Table 2B:** Top 5 enriched networks in downregulated proteins from lung adenocarcinoma samples (upregulated in squamous cell carcinoma).

**Network**	**Score**
Dermatological disease and conditions, Organismal injury and abnormalities	56
Cellular response to therapeutics, Cell death and survival, Cell to cell signaling and interaction	36
Cancer, Hematological diseases	21
Cell morphology, RNA post transcriptional modification, Cellular development	21
Cellular movement, cellular growth and proliferation, cellular development	19

## 2 Experimental design, materials and methods

### 2.1 Tissue collection and storage

This study was approved by the institutional review board (IRB) at All India Institute of Medical Sciences (AIIMS), New Delhi, India. The tumor tissues from 4 lung adenocarcinoma and 4 squamous cell carcinoma patients were obtained after surgical resection, histologically confirmed by an expert pathologist and stored at -80 °C till further analysis.

### 2.2 Tissue homogenization and protein isolation

All tissues were homogenized as described previously [1]. Briefly, 10 mg of tumor tissue was homogenized in 4% SDS using cell disperser (IKA works, Wilmington, NC) followed by sonication. The cell debris from tissue homogenates was removed by centrifugation at 14,000 rpm for 30 min at 4 °C. The cleared supernatant was transferred into a microfuge tube and the protein concentration determined using the BCA method.

### 2.3 Protein digestion and iTRAQ labelling

**Fig. 2:**
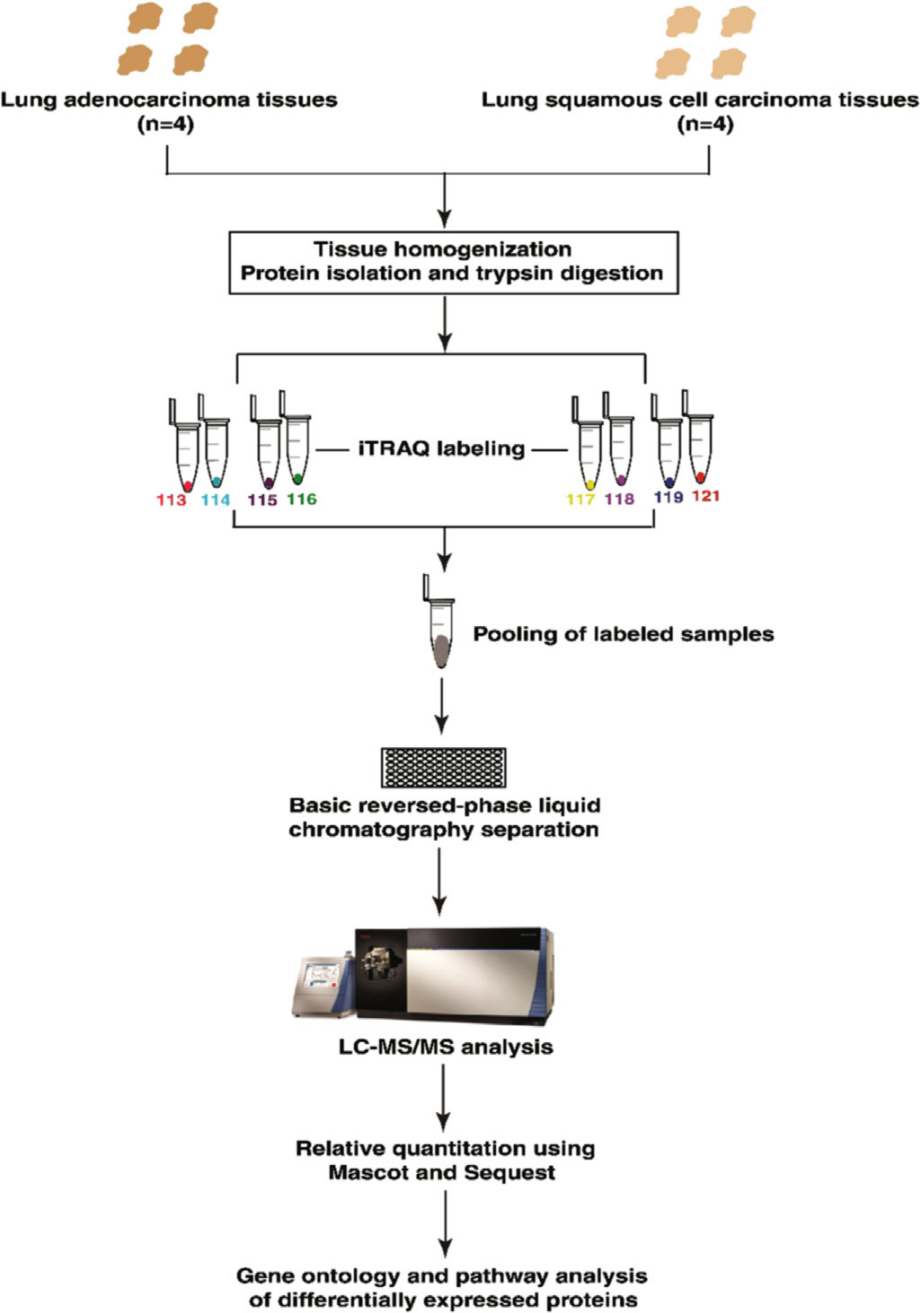
Workflow employed for quantitative proteomic analysis of lung adenocarcinoma and squamous cell carcinoma primary tissue samples. Proteins were extracted and quantified from lung adenocarcinoma and squamous cell carcinoma primary tissue samples. In-solution trypsin digestion of equal amount of proteins from each sample was performed and peptides were labeled with iTRAQ reagents. Pooled samples were fractionated using basic reversed phase liquid chromatography for proteomic analysis. All fractions were analyzed by mass spectrometer in triplicate.

In-solution trypsin digestion of samples from both conditions was carried out as described previously [2]. Equal amounts of cell lysate from all conditions were reduced using 5 mM dithiothreitol (DTT) and incubated at 60 °C for 45 mins. The reduced protein lysate was alkylated using iodoacetamide (IAA) (20 mM) and incubated for 15 mins in the dark at room temperature. To remove SDS, samples were buffer exchanged with 8 M urea followed by 50 mM TEABC to bring final concentration of SDS to 1 nano-mole. The samples were digested overnight with trypsin (Promega, Madison, WI) at an enzyme to substrate ratio of 1:20 at 37 °C. Peptides from each group were labelled using 8plex iTRAQ tags as per manufacturer׳s protocol. Peptides derived from adenocarcinoma samples were labelled with 113, 114, 115 and 116 labels while peptides from squamous cell carcinoma samples were labelled with 117, 118, 119 and 121 labels. All labels were pooled, dried and subjected to bRPLC fractionation. The workflow employed in quantitative proteomic analysis of lung adenocarcinoma and squamous cell carcinoma samples is represented in Fig. 2.

### 2.4 Basic reversed-phase liquid chromatography (bRPLC)

Briefly, 1 mL of bRPLC solvent A (10 mMTEABC pH 8.4, Sigma Aldrich) was used to resuspend peptide mixture. This mixture was fractionated by bRPLC chromatography on a XBridge C18, 5 μm, 250 × 4.6 mm column (Waters Corporation, Milford, MA) on an Agilent 1100 LC system with a flow rate of 1 mL/min by employing an increasing gradient of bRPLC solvent B (10 mMTEABC in 90% acetonitrile, pH 8.4). A total of 96 fractions were initially collected in 96- well plates with 0.1% formic acid added to each well. The fractions were then concatenated to 6 fractions and dried using speedvac.

### 2.5 LC-MS/MS analysis

Mass spectrometry analysis of 6 fractions was done in triplicate on Orbitrap Fusion mass spectrometer (Thermo Scientific, Bremen, Germany) interfaced with Proxeon Easy NanoLC system as described previously [3]. Peptides were reconstituted in 0.1% formic acid and they were loaded on the trap column (PepMap 100 C18, Nanoviper trap column, 75 µm x 20 mm, 3 µm) using 5% Acetonitrile, 0.1% Formic acid (Solvent A). Peptide separation was done on 75 µm ID x 25 cm in-house packed analytical column packed with Reprosil-Pur 120 C18-AQ, 1.9 µm (Dr. Maisch GmbH) using a step gradient of 8% to 22% solvent B (95% Acetonitrile, 0.1% Formic acid) for 70 min and 22% to 35% for 30 min at a flow rate of 280 nl/min. Total run time was 120 min and peptides were ionized by NanoFlex ion source maintained at 1850V. Orbitrap Fusion tribrid mass spectrometer connected to Proxeon Easy nLC 1000 system was used for LC-MS/MS analysis. Peptides were analyzed using data dependent top speed mode with synchronous precursor selection enabled for MS3 analysis. Total cycle time of 3 s was used for the analysis. Survey MS scan was collected in profile mode in Orbitrap mass analyzer using 350–1600 *m*/*z* mass range with 120,000 resolutions, 4*10^5^ AGC target and 50 ms injection time. Top most precursor ions were isolated using quadrupole mass filter with an isolation width of 0.7 Da and fragmented using collision induced dissociation with 35% normalized collision energy. MS2 spectra were acquired using Ion trap in rapid mode with 4000 AGC target and 100ms injection time. For MS3 analysis, top 10 precursor ions from MS2 spectra were isolated and fragmented using high energy collision induced dissociation (HCD) with 55% normalized collision energy. MS3 spectra were collected in Orbitrap mass analyzer with 50,000 resolutions, 1*10^5^ AGC target and 150ms maximum injection time. Dynamic exclusion was enabled with 40 s exclusion time. Lock mass of 445.12002 m/z from ambient air was enabled for mass recalibration during the run. Each fraction was analyzed in triplicate.

### 2.6 Data analysis

Proteome Discoverer (version 2.1.0.81) software suite (Thermo Fisher Scientific, Bremen, Germany) was used for MS/MS searches and protein quantitation. SEQUEST algorithm was used for database searches with NCBI RefSeq human protein database (Version 75). The search parameters included trypsin as the protease with maximum of 2 missed cleavages allowed; oxidation of methionine was set as a dynamic modification while static modifications included carbamidomethyl (alkylation) at cysteine and iTRAQ modification. Precursor mass tolerance was set to 10 ppm and fragment mass tolerance was set to 0.6 Da. The false discovery rate (FDR) was calculated by carrying out decoy database searches and peptides scoring better than 1% FDR score cut-off were considered for further analysis. Bioinformatics analysis of differentially expressed proteins from lung adenocarcinoma and squamous cell carcinoma samples was done to classify proteins based on subcellular localization and biological function. We performed classification based on annotations in the Human Protein Reference Database (HPRD; www.hprd.org) [4] which is in compliance with Gene Ontology (GO) standards. Pathway and network analysis of differentially expressed proteins was done using Ingenuity Pathway Analysis (IPA).
